# ERRATUM

**DOI:** 10.1590/0034-7167.20227506e05

**Published:** 2022-06-06

Article “The family of the child with special health care needs and their social relationships”, with number of DOI: https://doi.org/10.1590/0034-7167-2021-0031, published in the journal Revista Brasileira de Enfermagem, 75(Suppl 2): e20210031, on page 8:

Include:

**FUNDING**

This study was financed in part by the Coordenação de Aperfeiçoamento de Pessoal de Nível Superior - Brasil (CAPES) - Finance Code 001 and the Scientific Initiation Scholarship Program of the Federal University of Rio de Janeiro (PiBIC/UFRJ).

